# Diagnostic Value of the ^13^C Methacetin Breath Test in Various Stages of Chronic Liver Disease

**DOI:** 10.1155/2011/235796

**Published:** 2011-06-07

**Authors:** Hamizah Razlan, Nurhayaty Muhamad Marzuki, Mei-Ling Sharon Tai, Azhar-Shah Shamsul, Tze-Zen Ong, Sanjiv Mahadeva

**Affiliations:** ^1^Department of Medicine, Faculty of Medicine, University Kebangsaan Malaysia, 56000 Kuala Lumpur, Malaysia; ^2^Division of Gastroenterology, Department of Medicine, Faculty of Medicine, University of Malaya, 50603 Kuala Lumpur, Malaysia; ^3^Department of Community Health, Faculty of Medicine, University Kebangsaan Malaysia, 56000 Kuala Lumpur, Malaysia; ^4^Sentosa Kajang Medical Centre, 43000 Kajang, Selangor, Malaysia

## Abstract

The accuracy of the ^13^C-methacetin breath test (^13^C-MBT) in differentiating between various stages of liver disease is not clear. A cross-sectional study of Asian patients was conducted to examine the predictive value of the ^13^C-MBT in various stages of chronic liver diseases. Diagnostic accuracy of the breath test was determined by sensitivity, specificity, positive predictive value, negative predictive value, and area under the curve analysis. Seventy-seven patients (47 men/30 women, mean age 50 ± 16 years) were recruited. Forty-seven patients had liver cirrhosis (Child Pugh A = 11, Child Pugh B = 15, and Child Pugh C = 21), 21 had fibrosis, and 9 had chronic inflammation. The sensitivity and positive predictive value for liver fibrosis, cirrhosis (all stages), Child-Pugh A, Child-Pugh B, and Child-Pugh C were 65% and 56%, 89% and 89%, 67% and 42%, 40% and 40%, and 50% and 77%, respectively. Area under curve values for fibrosis was 0.62 (0.39–0.86), whilst that for cirrhosis (all stages) was 0.95 (0.91–0.99). The ^13^C-methacetin breath test has a poor predictive value for liver fibrosis but accurately determines advanced cirrhosis.

## 1. Background

The prognosis and management of chronic liver diseases usually requires knowledge of the stage and progression of disease. Liver biopsy, which is the gold standard in determining liver fibrosis and cirrhosis, is invasive and is associated with morbidity and mortality risks [1]. Furthermore, sampling variability can be found in 15%–30% of biopsies [2], and it is not used repeatedly to follow-up patients. Therefore, there is an increasing need for alternative noninvasive methods to diagnose liver fibrosis and cirrhosis.

Several quantitative liver function tests have been proposed to measure the functional hepatic mass [3]. However, these tests, although accurate, are cumbersome to perform and impractical in clinical settings. The Child-Pugh [4, 5] classification still remains the most widely used parameter of liver function. However, this classification does not strictly reflect the quantitative functional liver reserve, and its usefulness is limited by concomitant therapy with albumin, for example, and subjectivity on the degree of ascites and hepatic encephalopathy in an individual patient. 

Currently, several ^13^C breath tests based on the use of labeled substrates selectively metabolized within the liver are available to noninvasively assess hepatocellular function. Amongst the various substrates utilized to evaluate quantitative liver function, the ^13^C methacetin breath test (MBT) has shown to be most promising [6–8]. ^13^C Methacetin, a derivative of phenacetin, undergoes O-demethylation through the hepatic mixed oxidase system to acetaminophen and carbon dioxide. Compared with other ^13^C-labeled substrates, Methacetin is metabolized faster, rapidly cleared from the blood stream, safe, and cheap [9]. Several studies have demonstrated that the MBT reliably differentiates between healthy controls and patients with established cirrhosis [6, 10–12]. However, differentiating between patients with and without cirrhosis alone has limited value, as this can be performed reliably with routine clinical methods. A noninvasive tool to assess progression of liver disease in noncirrhotic (i.e., from chronic inflammation to fibrosis) and cirrhotic (from Child-Pugh grade A to C) patients would have greater utility in routine clinical practice. At present, many of the treatment/management algorithms for diseases such as the chronic viral hepatitis and nonalcoholic fatty liver disease require the confirmation of fibrosis/cirrhosis for either initiation or modification of therapy [13, 14]. To date, there is limited information on the MBT and its accuracy in differentiating between various grades of chronic liver disease of diverse aetiologies.

We aimed to assess the accuracy of the MBT in predicting liver fibrosis and grades of cirrhosis amongst patients with chronic liver diseases compared to the established clinical methods in our population.

## 2. Methods

### 2.1. Study Population

Consecutive patients from this multi-racial Asian population attending the gastroenterology outpatient and inpatient facilities of 2 institutions were prospectively recruited. All patients who had chronic liver disease of various aetiology and grades of liver disease were excluded. Informed consent from all patients and local ethical approval (The Ethical Committee, Medical Faculty, University Kebangsaan Malaysia and Medical Faculty, University Malaya) were obtained prior to the study. Patients with the following characteristics were excluded: those taking drugs with modulating capacity on P450 cytochrome activity, patients with portal vein thrombosis, heavy smokers (>10/day for >1 year), those with chronic lung diseases, and severe comorbid diseases. 

### 2.2. Confirmation of Liver Disease

All patients had an established diagnosis of liver disease based on a combination of clinical, biochemical and radiological features. Noncirrhotic patients were diagnosed by histology. The liver histology was classified by an experienced pathologist according to the Scheuer classification [15]. The classification categorizers 5 different stages of fibrosis: stage 0—no fibrosis, stage 1—enlarged fibrotic portal tracts, stage 2—periportal or portal-portal septa, stage 3—fibrosis with architectural distortions, and stage 4—cirrhosis. Liver biopsies were performed in all cases at least 2 weeks (ranging from 14–23 days) before having the MBT. Child-Pugh grade A disease was diagnosed in patients with histological confirmation of cirrhosis but no features of hepatic decompensation. Advanced cirrhosis (Child-Pugh grade B and C) was diagnosed in individuals with definite portal hypertension, clinical features of decompensation and/or radiological imaging.

### 2.3.  ^13^C Methacetin Breath Test

^13^C-MBT was performed after at least 8 hours of fasting. A baseline breath sample was taken to evaluate the amount of ^13^C present at baseline (international standard ratio = 0.1%). The patients were then given 75 mg of ^13^C Methacetin (99%  ^13^C, Cambridge Isotope Laboratories, Andover, Mass, USA) dissolved in 50 ml of tea to drink. Breath samples were obtained by slow expiration through a tube into a breath sample bag, after a deep inspiration. Breath samples were collected at ten-minute intervals for the first hour and at twenty-minute intervals for up to 120 minutes after substrate administration. All subjects were required to be at rest and without drinking and eating for the duration of the test. The 13CO2/12CO2 isotope ratio in the breath samples was analyzed by nondispersive isotope-selective infrared spectrometry (Wagner Analysentechnik, Bremen, Germany) [16]. The *δ* values obtained were related to the baseline *δ* values. The percentage of ^13^C exhaled was calculated assuming a CO2 production rate of 5 mmol/min m^2^. The results were expressed as the cumulative percentage (%) of the administered dose of ^13^C recovered over time, which corresponded to the administered dose of ^13^C per hour. 

### 2.4. Study Analysis and Statistics

Discriminatory ability of the 13C MBT was quantified by using an area under the receiver operating curve [17]. The MBT value that correctly classified all subjects had an area of 1.0 (perfect discrimination), and the value with no discriminatory power had an area of 0.5 or less. AUC values of 0.7-0.8 and >0.8 were considered to represent reasonable and good discrimination [18] respectively. The sensitivity, specificity, positive predictive value (PPV) and negative predictive value (NPV) were additionally determined to assess the accuracy of predetermined ^13^C MBT “cutoff ” values.

^13^C breath test data were expressed as means with standard deviation, and comparisons between patients with various liver diseases were performed using the Mann-Whitney *U* test (i.e., nonparametric data). Statistical significance was assumed at a *P* value of <.05.

## 3. Results

Seventy-seven people (47 men/30 women, mean age 50 ± 16 years) were recruited for the study between March 2006 to March 2007. The majority of patients had chronic (viral) hepatitis B (32.5%) and C (26.0%) infection. Forty-seven patients had liver cirrhosis and twenty one patients had fibrosis at various stages..Further 9 patients had chronic inflammation of various aetiology (Table 1). Among patients with cirrhosis, the severity of liver disease were as follows: Child Pugh A *n* = 11, Child Pugh B *n* = 15, and Child Pugh C *n* = 21. Stages of fibrosis in noncirrhotic patients were as follows: stage 1 *n* = 10, stage 2 *n* = 7, stage 3 *n* = 3, and stage 4 *n* = 1 (Table 1).

Mean cumulative recovery percentages (metabolic capacity) at both 40 and 120 minutes were compared between patients with various stages of liver disease (Table 2). At 40 minutes of metabolic capacity, MBT values were significantly lower in patients with cirrhosis compared to those without (0.25 ± 0.22 versus 0.77 ± 0.19, *P* < .0001). MBT values were significantly different between all grades of liver cirrhosis (Table 2). However, in noncirrhotic patients (*n* = 30), no significant differences in MBT values were noted between cases with and without fibrosis (0.72 ± 0.17 versus 0.80 ± 0.25, *P* = .33). Similar results were obtained for cumulative recovery percentages at 120 minutes (Table 2).

**Figure 1:**
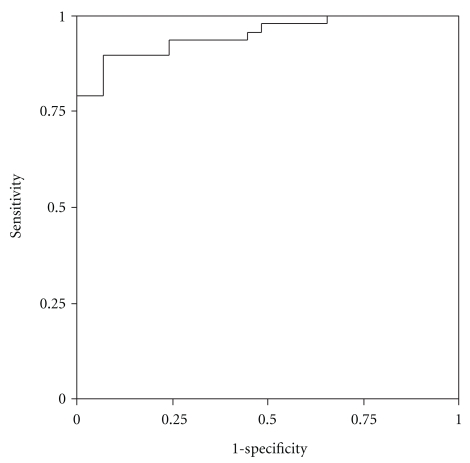
Receiver operating characteristic curve analysis of cumulative percent oxidation of methacetin at 40 minutes in patients with and without cirrhosis.

**Figure 2:**
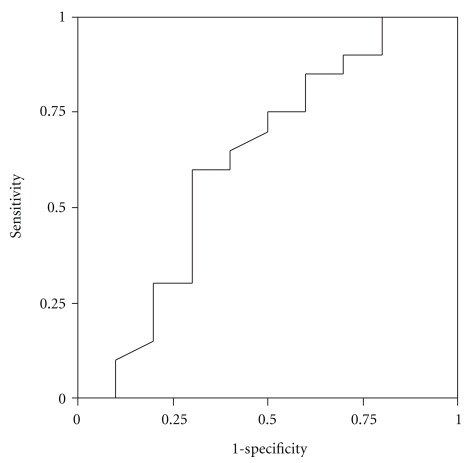
Receiver operating characteristic curve analysis of cumulative percent oxidation of methacetin at 40 minutes in patients with and without fibrosis (i.e., noncirrhotic).

Discriminatory ability of the MBT was assessed based on the 40 minute cumulative recovery percentages (Table 3). The MBT was able to discriminate well between patients with and without cirrhosis (AUC 0.91, 95% CI = 0.82–0.99) and between Child-Pugh C cirrhosis and those without (AUC 0.91, 95% CI = 0.82–0.99), as illustrated in Figure 1. However, it had poor discriminatory power for Child-Pugh A (AUC 0.47) and less than reasonable differentiation for Child-Pugh B (AUC 0.69) and liver fibrosis (AUC 0.67). The latter is shown as an example in Figure 2. 

The accuracy of predetermined “cut-off ” MBT values for various stages of liver disease are highlighted in Table 3. Briefly, predicted “cut-off ” values of the MBT for liver fibrosis had a 65% sensitivity and a PPV of 56%, whilst sensitivity and PPV for cirrhosis (all stages) were 89% and 89% respectively. Sensitivity and PPV for Child-Pugh A, B, and, C were 67%, 40%, and 50% and 42%, 40%, and 77%, respectively (Table 3). Conversely, specificity and NPV of the MBT were reasonable: fibrosis 82% and 87%, cirrhosis (all stages) 83% and 83%, Child-Pugh A cirrhosis 83% and 93%, Child-Pugh B cirrhosis 85% and 85%, and Child-Pugh C cirrhosis 95% and 84% (Table 3). 

None of the patients in the study sustained an adverse reaction to methacetin.

## 4. Discussion

The MBT has been purported as a suitable alternative to standard clinical methods in assessing liver function such as the Child-Pugh score/grade [19, 20]. However, clinicians managing patients with chronic liver disease require a diagnostic measurement/modality that reliably characterises the natural history of chronic liver disease, that is, from nonfibrotic inflammation to fibrosis and eventually to the various stages of cirrhosis. In previous studies, the ^13^C-MBT has been demonstrated to reliably differentiate between healthy adult controls and patients with liver cirrhosis [19, 20]. A few recent publications have also demonstrated that liver fibrosis can be predicted in patients with chronic viral hepatitis [21, 22]. Dinesen et al. compared the diagnostic accuracy of using ^13^C-MBT with several noninvasive tests like the APRI, AAR, and Fibroindex and found that the ^13^C-MBT was more reliable in predicting advanced fibrosis and cirrhosis in patients with chronic hepatitis C [22]. Lalazar et al. also demonstrated that by using a continuous automatic molecular correlation spectroscopy BreathID, an accurate detection of liver inflammation and fibrosis was obtained on patients with chronic hepatitis C with normal ALT levels [23]. However, differentiation between stages of chronic liver disease and cirrhosis has not been established. 

In this pragmatic study of Asian patients with chronic liver disease of various aetiologies, we have demonstrated that the MBT was only of value in discriminating between cases with and without cirrhosis, particularly in those with more advanced cirrhosis (Child-Pugh B and C). The MBT could not reliably differentiate between patients with and without fibrosis in noncirrhotic patients. This observation, as assessed by the AUC, was further supported by differences in the mean cumulative oxidation capacity values. Additionally, predicted values of the MBT for various stages of liver disease were shown to have a low sensitivity and PPV for hepatic fibrosis and some stages of cirrhosis, but a reasonable specificity and NPV. 

Sensitivity, specificity, PPV, and NPV of the MBT for predicting cirrhosis were similar to a recent study of 96 German patients with chronic hepatitis C (sensitivity 92.6%, specificity 84.1%, PPV 69.4%, and NPV 96.7%) but very different in the case of hepatic fibrosis [22]. Similarly, our results are consistent with a previous Spanish study of 48 patients with various stages of chronic liver disease, which demonstrated that the MBT was sensitive at detecting cirrhosis but poor at differentiating between chronic hepatitis and early stages of cirrhosis [8]. However, these results have been challenged by a few recent studies that have demonstrated reasonable differentiation between stages of cirrhosis in patients with mostly hepatitis C-related liver disease [19, 24].

The most useful expression of MBT kinetic parameters from a diagnostic perspective is the cumulative percent oxidation at a particular time period. In the literature, there has been some variation to the precise cumulative recovery time selected to determine hepatic function. Cumulative recovery times of 10 minutes [8], 15 minutes [21], 30 minutes [12, 19], and 60 and 120 minutes [20] have been reported in various populations to be the ideal predictive time of hepatic function. Variations in gastric emptying time are believed to account for some of these differences. However, it is now suggested that cumulative recovery periods beyond 60 minutes are not necessary, as the results for cumulative recovery are best seen in periods <60 minutes [21]. In view of these data, the oxidation period of 40 minutes that was used in this study, based on the IRIS manufacturer's recommendation, seems reasonable and appropriate. Furthermore, the hepatic oxidation of MBT takes account of body surface area and has been shown to be reproducible in non-Western ethnic groups as well [25].

A possible explanation for the poor diagnostic value of the MBT in our patient sample may have been due to the aetiology of liver disease in this region. Most of the studies that have examined the diagnostic ability of the MBT have been performed in Western patients with chronic hepatitis C. In the Asia-Pacific region, hepatitis B remains the commonest cause of chronic liver disease [13], and this was reflected in our patient sample. Whilst hepatic function in cirrhosis is fairly similar regardless of aetiology, it is possible that variations in noncirrhotic liver disease hepatocyte function due to different aetiologies may account for the MBT results observed.

## 5. Conclusions

We conclude that the ^13^C-MBT is useful in the diagnosis of advanced cirrhosis in Asian patients with chronic liver disease. However, this study has demonstrated that the MBT does not reliably differentiate between noncirrhotic and various stages of cirrhosis in our group of patients, which limits its application in routine clinical practice at this stage. Further studies are required to determine if modifications of the MBT or substrate are required to improve diagnostic ability in Asian patients with chronic liver disease.

##  Conflict of Interests

None of the authors involved in this study have any competing interests to declare with respect to the publication of this paper.

##  Authors' Contributions

H. Razlan, T.-Z. Ong and S. Mahadeva designed the study, analysed and interpreted the data, and drafted the paper. N. M. Marzuki and M.-L. S. Tai performed all data collection. A.-S. Shamsul contributed to statistical analysis. All authors reviewed and approved final version of the paper.

## Figures and Tables

**Table 1:** Baseline characteristics of the studied population (*n* = 77).

Gender (male/female)	47 (61%)/30 (39%)
Age (years)	50 ± 16
Ethnic groups	
Malay/Chinese/Indian	27 (35%)/37 (48%)/13 (17%)

Aetiology^#^	
HBV	25 (32.5%)
HCV	20 (26.0%)
NAFLD	9 (11.7%)
Cryptogenic	7 (9.1%)
Myelofibrosis	1 (1.3%)
Alcohol	12 (15.6%)
PBC/AIH	3 (3.8%)

Cirrhosis/non-cirrhosis	
Child-Pugh class A	11 (23.4%)
Child-Pugh class B	15 (31.9%)
Child-Pugh class C	21 (44.7%)

Stages of fibrosis	
Stage 0	9 (30.0%)
Stage 1	10 (33.3%)
Stage 2	7 (23.3%)
Stage 3	3 (10.0%)
Stage 4	1 (3.33%)

^#^Abbreviations: HBV: chronic hepatitis B; HCV: chronic hepatitis C; NAFLD: non alcoholic fatty liver disease; PBC: primary biliary cirrhosis; AIH: autoimmune hepatitis.

**Table 2:** Mean values of the cumulative percent oxidation of the ^13^C-MBT at 40 and 120 mins in various stages of chronic liver disease.

Stages of liver disease	*n*	Cum. 40 mins^a^	Mean difference (95% CI)	*P*	Cum. 120 mins^b^	Mean difference (95% CI)	*P*
Fibrosis	21	0.72 ± 0.17	−0.07	.33	0.85 ± 0.14	−0.09	.11
Nonfibrosis/ inflammation	9	0.80 ± 0.25	(−0.24 to 0.08)		0.94 ± 0.19	(−0.22 to 0.03)	
Cirrhosis	47	0.25 ± 0.22	−0.51	<.0001	0.43 ± 0.26	−0.47	<.0001
Noncirrhosis	30	0.77 ± 0.19	(−0.61 to −0.41)		0.89 ± 0.15	(−0.57 to −0.36)	
CPA* cirrhosis	12	0.45 ± 0.18	−0.31	<.0001	0.64 ± 0.19	−0.25	<.0001
noncirrhosis	29	0.76 ± 1.89	(−0.44 to −0.18)		0.89 ± 0.15	(−0.36 to −0.13)	
CPB^#^ cirrhosis	15	0.26 ± 0.17	−0.41	<.0001	0.46 ± 0.23	−0.36	<.0001
CPA/noncirrhosis	41	0.67 ± 0.23	(−0.54 to −0.28)		0.82 ± 0.20	(−0.49 to −0.24)	
CPC^∗∗ ^cirrhosis	21	0.12 ±0.19	−0.44	<.0001	0.28 ± 0.22	−0.45	<.0001
Non-CPC cirrhosis	56	0.56 ± 0.28	(−0.57 to −0.30)		0.72 ± 0.26	(−0.57 to −0.31)	

*Child-Pugh A, ^#^Child-Pugh B, and **Child-Pugh C.

^a^Normal range: 0.90–1.20.

^b^Normal range: 0.80–1.10.

**Table 3:** Predictive value of the ^13^C-MBT in various stages of liver disease based on the cumulative recovery of oxidation at 40 minutes.

	Sensitivity (95% CI)	Specificity (95% CI)	PPV (95% CI)	NPV (95% CI)	AUC (95% CI)
Fibrosis	65 (44–86)	82 (72–92)	56 (36–76)	87 (78–96)	0.62 (0.39–0.86)
Cirrhosis (all stages)	89 (80–98)	83 (69–97)	89 (80–98)	83 (69–97)	0.95 (0.91–0.99)
